# Posterior Urethral Valves in Neonate

**Published:** 2013-07-01

**Authors:** Vivek Gharpure

**Affiliations:** Department of Pediatric Surgery, Children's Surgical Hospital 13, Pushpanagari, Aurangabad, 431001, India

 (This section is meant for residents to check their understanding regarding a particular topic)

## Questions

1. Demography of PUV?
2. Prenatal diagnosis of PUV?
3. Prenatal interventions for PUV?
4. Neonatal management of PUV?
5. Diagnosis of PUV?
6. Definitive intervention in PUV in neonate?
7. Early diversion in PUV?
8. What complications are associated with valve ablation/ fulguration?
9. Describe options for urinary diversion in PUV.
10. Compare outcomes in vesicostomy and electrofulguration in PUV.
11. Discuss follow up care of PUV.

## Answers

1. PUV is the commonest etiology of urinary tract obstruction in the neonate. Incidence reported 1:5000 to 1: 8000. It is the commonest cause of chronic kidney disease secondary to urinary obstruction in children. A population based study found the incidence to be 2.48 (2.14 – 2.81) per 10,000 live births [1].
2. Prenatal ultrasound scanning has increased detection rate of PUV. In a population-based study, prenatal diagnosis rate was 46.9% [1].
  Sonographic features of PUV include:
  1. Thick-walled bladder
  2. Bilateral hydronephrosis/ hydroureter (may be unilateral)
  3. Scanty liquor
  4. Keyhole sign- dilated bladder and posterior urethra
  5. Echo-bright kidneys
  Differential diagnoses include:
  1. Prune belly syndrome
  2. Megaureter
  3. Megacystis-microcolon syndrome
3. Prenatal interventions like vesico-amniotic shunt/ vesicocentesis have been done for many years. The rationale is that early drainage of obstructed system will allow improvement in renal function, improve survival and prevent lung damage.
Meta-analysis by Clark et al [2] demonstrated improved survival or renal function in those patients who underwent drainage, and who were considered to have poor prognosis. But among survivors, prenatal drainage did not alter renal function status.
Early detection and Oligohydramnios are associated with poor outcome.
Prenatal diagnosis has not improved long term outcomes.
PLUTO trial did not answer questions on benefit of prenatal intervention in bladder outflow obstruction [3]. However, Cortes-Osario et al. found that early decompression of obstructed urinary tract in the fetus had a protective role and prevented kidney damage [4].
4. This will depend upon whether the baby has been prenatally diagnosed/suspected to have PUV.
After delivery, bladder is catheterized with 6 Fr. feeding tube and antibiotics are administered. Patient is closely observed for hydration and electrolyte balance. Blood urea and creatinine are measured. Urinary creatinine may be measured. Urinary volume, specific gravity, osmolality can be measured. Some patients may develop high output failure and will need appropriate fluid management.
Babies may present in neonatal period with retention of urine, urosepsis, failure to thrive, dehydration, acidodis, pyuria or with vague symptoms and abdominal signs. These will need fluid resuscitation, catheterization, administration of antibiotics, urine c/s and monitoring of renal function.
Priority in all patients with PUV is stabilization. Once the bladder is catheterized, it is no longer a surgical emergency.
Rarely, it may not be possible to catheterize the bladder. Some of them may require suprapubic insertion of a catheter. If kidneys fail to drain despite adequate bladder drainage, percutaneous pyelostomy may be required, especially if the kidney is infected.
5. Voiding cysto-urethrogram (VCUG or MCU); is the mainstay of diagnosis for PUV. Ultrasound scan may demonstrate enlarged/thick walled bladder, dilated urethra, dilated kidneys and ureters, bladder diverticulae. Urinary ascites/urinoma may be seen on USG Scan. Contrast enhanced sonourethrography has been compared with conventional MCU and has been found reliable [5].
At this stage, isotope scans are not necessary
VCUG shows dilated/small bladder, presence of reflux, sacculations, trabeculations of bladder, dilatation of posterior urethra, bladder neck hypertrophy.
Micturition films must be obtained after removing catheter. This requires patience and quick action by radiologist.
6. Endoscopic valve ablation remains the definitive intervention in PUV. Valves are destroyed by electrofulguration. Earlier cutting loops were used; these days, bugbee electrode is preferred.
Some surgeons prefer to use cold knife. Mohan’s valvotome can be used in some neonates [6]. Soliman has used Fogarty catheter and 7.5 Fr. cystoscope for valve ablation [7]. Valves were destroyed by traction. They found satisfactory results in 93% (13/14) patients.
Valves are incised at 5 o’clock and 7 o’clock position. The role of bladder neck incision is controversial.
Following valve ablation, bladder is catheterized for 48 hours; IV fluids, antibiotics are administered. Patients are monitored for high output failure.
7. Preterm babies, small neonates may not be suitable for endoscopic valve ablation. These babies can be managed with indwelling bladder catheter till they are able to undergo cystoscopic ablation. Failing this, cutaneous vesicostomy provides for adequate low-pressure bladder drainage. Patients can undergo valve ablation and vesicostomy closure at a later date
Some patients with enormously dilated ureters and poor drainage may benefit by ureterostomy. A high loop ureterostomy provides better drainage than low ureterostomy. Different types of ureterostomies are practiced, depending upon surgeon’s preference.
Supravesical drainage has not been conclusively shown to improve outcomes and is at best a temporizing measure in those babies with fragile kidneys.
8. Valve ablation/fulguration can be associated with significant morbidity in the neonate as neonatal urethra is small and prolonged instrumentation can cause damage.
Strong current, excessive destruction of tissue can lead to stricture formation.
Intentional or accidental incision of bladder neck is associated with urinary incontinence.
Retention of urine after removal of catheter is commonly seen and requires further catheterization and a second trial of micturition
Hematuria, urinary tract infections are not common [8].
Other complications are urinary extravasation, bladder injury are rare.
9. a. Cutaneous vesicostomy:
This is the simplest urinary diversion. It can be done under local anesthesia; it is possible to do it in the NICU in a preemie, or an unstable neonate. Lapides vesicostomy is preferred. It is a tubeless diversion. It is important to mobilize the bladder dome in wound, to avoid prolapse and excise a triangle of rectus sheath to prevent stenosis. Low-pressure drainage allows the kidneys to decompress. Vesicostomy can be closed whenever conditions are favorable. Vesicostomy closure is a simple procedure.
b. Loop ureterostomy:
This is technically more difficult than vesicostomy, but provides direct drainage of kidney. This should be done only when ureters are dilated and tortuous; otherwise the ureter cannot be mobilized to surface and may develop ischemic necrosis. Bilateral flank incisions are made, muscles incised and ureters are identified in the retroperitoneum. A loop of ureter is brought to surface. Two limbs are sutured to each other. Muscle of ureter is sutured to abdominal wall muscles. Ureter is opened and mucosa is sutured to skin. A retention bridge is left for two weeks.
c. End ureterostomy:
This helps to normalize ureter size and drains kidneys, if ureters have normal motility. This can be done when ureters are dialated and tortuous. In doubtful functioning kidneys, this allows clinical assessment of urine output from each kidney. Incision is similar but slightly higher to that used for herniotomy. Ureter is identified in the retroperitoneum, and divided. Upper end is brought out separately, and a spout is made. Main incision is closed.
d. Ring ureterostomy:
This theoretically allows some urine to drain in bladder, but this is a technically demanding procedure, and not suitable for a sick neonate.
e. Pyelostomy/Nephrostomy:
  These require insertion of a catheter in the renal pelvis. Catheters are associated with chronic infection, irritation, foreign body reaction, thickening of pelvis and ureters and difficult subsequent reconstruction.
10. Narasimhan et al in a prospective study over 6 years compared vesicostomy and fulguration in 45 consecutive neonates with PUV. Patients were analyzed for renal function and somatic growth. They did not find significant difference in two treatment modalities [9].
11. All patients are followed for improvement of renal function, somatic growth, resolution of vesicoureteric reflux (if present), monitored for urinary tract infection. Urodynamics evaluation is done when the child is older. A check MCU or cystoscopy is recommended 3 months following initial cystoscopy.
Alpha- adrenergic blockers have been used by Abraham et al in PUV patients. They used Terazosin in 42 boys, and post void residue was reduced in 40 patients [10].
Patients also need Vit. D-3 supplements.
Vesicoureteric reflux should be surgically corrected if it persists beyond 3-5 years, or earlier if patient develops repeated breakthrough infections or develops new renal scars.
Some patients may require bladder augmentation.
Patients should be monitored till they attain puberty, as some patients can develop end stage renal disease at puberty.

## Footnotes

**Source of Support:** Nil

**Conflict of Interest:** None

